# Addressing the Challenges of Tuberculosis: A Brief Historical Account

**DOI:** 10.3389/fphar.2017.00689

**Published:** 2017-09-26

**Authors:** Hussam W. Al-Humadi, Rafal J. Al-Saigh, Ahmed W. Al-Humadi

**Affiliations:** ^1^Department of Pharmacology and Toxicology, Pharmacy College, University of Babylon, Babylon, Iraq; ^2^Laboratory of Pharmacology, Medical School, National and Kapodistrian University of Athens, Athens, Greece

**Keywords:** tuberculosis, history, treatment, anti-TB drugs, multidrug resistance, pharmaceutical innovation

## Abstract

Tuberculosis (TB) is a highly contagious disease that still poses a threat to human health. *Mycobacterium tuberculosis* (MTB), the pathogen responsible for TB, uses diverse ways in order to survive in a variety of host lesions and to subsequently evade immune surveillance; as a result, fighting TB and its associated multidrug resistance has been an ongoing challenge. The aim of this review article is to summarize the historical sequence of drug development and use in the fight against TB, with a particular emphasis on the decades between World War II and the dawn of the twenty first century (2000).

## Introduction

Tuberculosis (TB) is a very old infectious disease, caused by *Mycobacterium tuberculosis* (MTB) (Dye and Williams, 2010). It's still the second most frequent cause of death in the world (WHO, 2014), reaching up to 10 million new cases every year (Dye and Williams, 2010); more interestingly, latent cases represent one third the world's population (WHO, 2014), with 10% of latent TB cases to progress to active infection (Selwyn et al., 1989), especially in diabetic or human immunodeficiency virus (HIV) positive patients, or those undergoing an immunotherapy (Barry et al., 2009). Active TB is characterized by chronic cough with bloody sputum, night sweats, fever and weight loss, while other organs (apart from the lungs) can be infected and cause a wide range of symptoms (Dolin Gerald et al., 2010).

TB bacilli are spread with the droplets of respiratory secretions that are associated with cough or sneezing of the infected person. The MTB can then invade and replicate within the endosomes of the pulmonary alveolar macrophages (Houben et al., 2006; Kumar et al., 2007) leading to clinically active disease in about 10% of cases (Dye et al., 1999; WHO, 2009), while further growth of the remaining cases can be arrested by a competent immune response. However, in those with arrested cases, the bacilli are completely eradicated in about 10% of the individuals, with the remaining 90% entering a dormant or latent state in which there is a containment of the infection. As pathogens escape from the microbicidal action of the host immune cells (phagosome-lysosome fusion; MHC class I, class II, and CD1 molecules antigens; nitric oxide and other reactive nitrogen intermediates), latent TB and the dormant bacilli are reactivated with any serious disruption (decline) in the host immune state (HIV infection, diabetes mellitus, renal failure, chemotherapy and immunosuppressive therapy, malnutrition, etc.) that occurs (Dye et al., 1999; Corbett et al., 2003; Frieden et al., 2003; Wells et al., 2007; Dooley and Chaisson, 2009; WHO, 2009).

The unique clinical manifestations of MTB are attributed to the high lipid content of this pathogen (Southwick, 2007); the latter has an outer membrane lipid bilayer (Niederweis et al., 2010) and therefore, hematogenous transmission can also spread infection to more distant sites, such as peripheral lymph nodes, the kidneys, the brain, and even the bones (Harries, 2005; Herrmann and Lagrange, 2005; Kumar et al., 2007).

The public health challenge of TB has been managed by a number of drugs and treatment strategies over the years, but this challenge has always been much bigger in certain parts of the world. The spreading of the HIV infection has been a major factor in managing the TB challenge, and so has been the increasing resistance of MTB strains to the high efficacy first line anti-TB drugs (Table 1; WHO, 2009) which leads to the growing incidences of drug resistant strains: multiple drug resistant (MDR) and extensively drug resistant (XDR). These strains pose a significant threat, especially for immunocompromised patients who are significantly less likely to recover without the assistance of effective drugs. Other factors that may contribute in disease progression include poverty, population expansion, active transmission in overcrowded places (hospitals, prisons, and other public places), migration of individuals from high-incidence countries due to wars or famine, drug abuse, social decay, homelessness (Frieden et al., 2003; Hill et al., 2004; Mathema et al., 2008) and technical problems like poor quality of detection, in addition to health status (old age, malnutrition, and medical conditions that compromise the immune system) (Corbett et al., 2003; Frieden et al., 2003; Wells et al., 2007; Dooley and Chaisson, 2009).

**Table 1:** Classification of anti-tuberculosis (anti-TB) drugs according to WHO (2010).

**Lines**	**Grouping**	**Drugs**	
First-line anti-TB drugs	Group 1 (oral)	Isoniazid (H/INH)	
		Rifampicin/rifampin (R/RIF)	
		Pyrazinamide (Z/PZA)	
		Ethambutol (E/EMB)	
		Rifapentine (P/RPT)	
		Rifabutin (RFB)	
Second-line anti-TB drugs	Group 2 (injectable)	Aminoglycosides	Streptomycin (S/STM)
			Kanamycin (KM)
			Amikacin (AMK)
		Polypeptides	Capreomycin (CM)
			Viomycin (VIM)
	Group 3 (oral and injectable; fluoroquinolones)	Ciprofloxacin (cfx)	
		Levofloxacin (lfx)	
		Moxifloxacin (mfx)	
		Ofloxacin (OFX)	
		Gatifloxacin (GFX)	
	Group 4 (oral)	Para-aminosalicylic acid (PAS)	
		Cycloserine (DCS)	
		Terizidone (TRD)	
		Ethionamide (ETO)	
		Prothionamide (PTO)	
		Thioacetazone (THZ)	
		Linezolid (LZD)	
Third-line anti-TB drugs	Group 5 (oral and injectable)	Clofazimine (CFZ)	
		Linezolid (LZD)	
		Amoxicillin plus clavulanate (AMX/CLV)	
		Imipenem plus cilastatin (IPM/CLN)	
		Clarithromycin (CLR)	

Furthermore, the unusual structure and chemical composition of the MTB cell wall (which hinders the entry of drugs and leads to drugs resistance) (Brennan and Nikaido, 1995) as well as the capability of the MTB cell to lie dormant at a low metabolic rate, in a deep location in pulmonary cavities or inside solid material that makes antibiotic penetration difficult. Finally, expensive, long-term therapy, disturbed therapeutic regimens, dosage variance and irregularity in follow up, form additional challenges for an effective TB management (Lawn and Zumla, 2011).

The aim of this review article is to summarize the historical sequence of drug development and use in the fight against TB, with a particular emphasis on the decades between World War II and the dawn of the twenty first century (2000).

## Historical sequences in management of TB: before world war II

There is evidence of TB being present in humans since antiquity (Lawn and Zumla, 2011). MTB has been detected in the remnants of a bison in Wyoming that lived 17,000 years ago (Rothschild et al., 2001), while researchers have found tubercular decay in the spines of Egyptian mummies (3000–2400 BC) (Zink et al., 2003), and genetic studies suggested TB was present in America since around 100 AD (Konomi et al., 2002). In Europe, TB had begun to rise between seventeenth and nineteenth century, in which it reached a peak level and caused about 25% of all deaths (Bloom, 1994). At that time, several measures had been taken including the improvement of life style and the encouragement of the infected people to enter sanatoria (McCarthy, 2001). However, 50% of those who entered sanatoria died within 5 years (McCarthy, 2001).

On 24 March 1882, MTB was identified and described by Robert Koch; he was later honored with the Nobel Prize (1905) for this discovery (Nobel Foundation, 2014), the “TB World Day” was established on that date. Koch didn't pay attention for the similarity between bovine and human TB, therefore, the recognition for TB-infected milk as a way of TB transmission was delayed until the invention of the pasteurization process, that reduced it dramatically. Koch announced a glycerin extract of the TB bacilli as a “remedy” for TB in 1890, calling it “tuberculin.” Even though, it was not effective, it was later adapted as a screening test for the presence of latent TB (Waddington, 2004).

In 1906, Albert Calmette and Camille Guérin achieved the first genuine success in immunization against TB by using attenuated bovine-strain TB. It was called the “bacille Calmette-Guérin” (BCG). This vaccine was first used on humans in 1921 in France (Bonah, 2005), but the vaccine got widespread acceptance in the US, Great Britain, and Germany only after World War II (Comstock, 1994).

**Figure 1:**
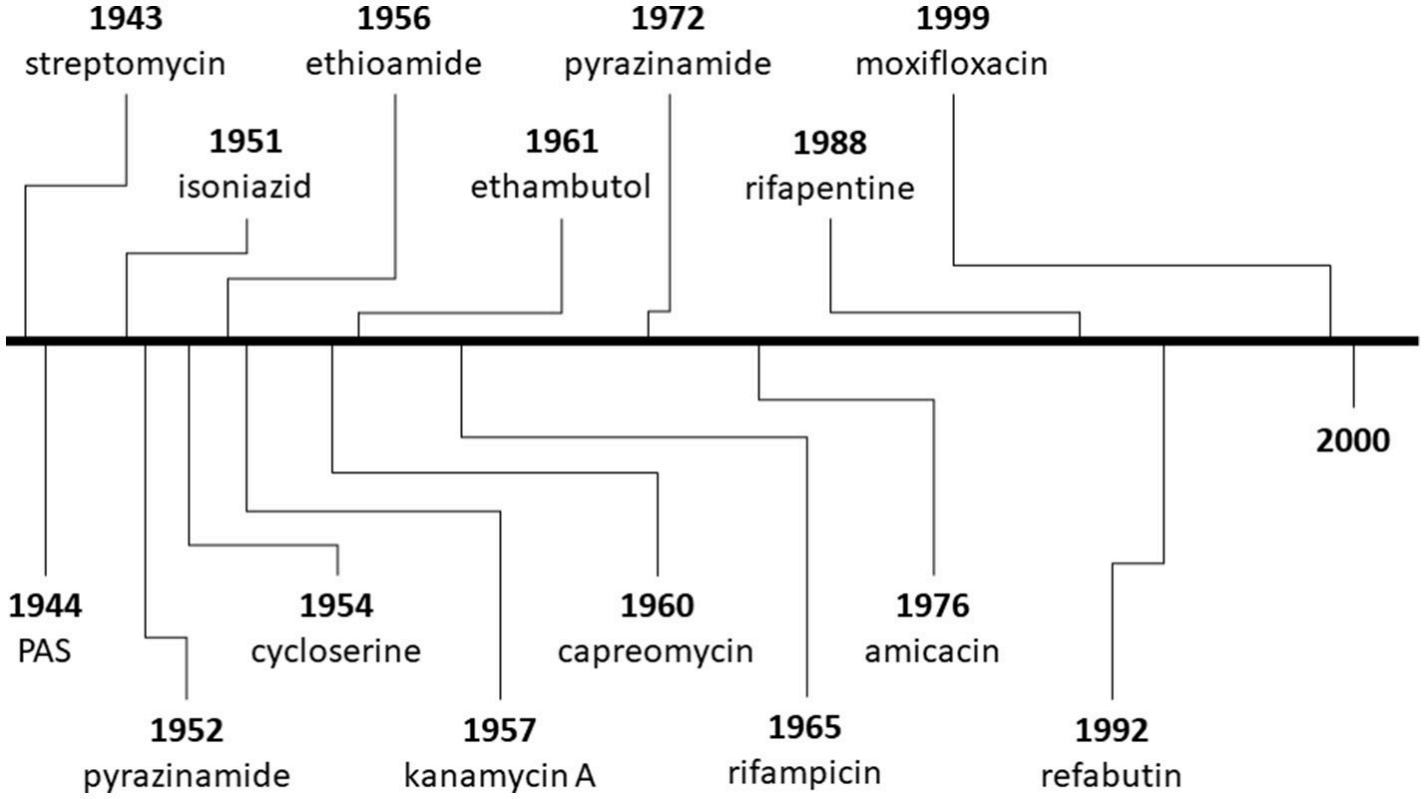
Timeline of the discovery of major compounds used for the treatment of tuberculosis (TB) from the Second World War until 2000.

The discovery of penicillin initiated the war against various infectious microorganisms, and has set the basis for a greater motivation to discover other antibacterial and antimicrobial compounds for overcoming diseases like TB. The success of penicillin during World War II pushed researchers to study other molds (Aminov, 2010), one of them being Streptomyces griseus, found in chickens; as a result, streptomycin was successfully purified in 1943 and used as an anti-TB therapy in 1945 (Schatz et al., 1944; Kerantzas and Jacobs, 2017). Unfortunately, the overuse of streptomycin led to the development of drug-resistance (Kerantzas and Jacobs, 2017), but the end of World War II saw major developments in pharmacology been established, and a number of drugs being developed and used against TB (Figure 1; Schatz et al., 1944; Wassersug, 1946).

## Historical sequences in management of TB: after world war II

In 1946, the Medical Research Council (MRC) TB Unit in the UK was established, and a clinical study designed for comparing streptomycin with bed rest vs. bed rest alone (Marshall, 1949) was launched. As expected, high clinical improvement was seen in streptomycin with bed rest in comparison to bed rest alone, however, a greater improvement was seen in the first 3 months, and many patients deteriorated later on due to the emergence of streptomycin resistance (Kerantzas and Jacobs, 2017).

Better results followed with the development of para-aminosalicyclic acid (PAS), which was an oral agent (unlike streptomycin) and could be used in combination with streptomycin (Lehmann, 1946; British Medical Journal, 1950; Fox et al., 1999; Williams, 2009). In 1950s, several anti-TB drugs with different mechanisms of action were discovered and developed, including PAS, isoniazid, pyrazinamide, cycloserine and kanamycin (Figure 1, Table 2). In 1951, streptomycin plus isoniazid were introduced as a TB therapy (Fox et al., 1999), while rifampicin (in 1960) allowed the shortening of TB therapy to 9 months when given with isoniazid, and to 6 months when given with pyrazinamide (American Thoracic Society, 2003). By the 1970s, five antibiotics were available against TB (Figure 1). Afterwards, the MRC TB Unit developed the current short-course therapeutic regimen (isoniazid, rifampicin, pyrazinamide and ethambutol) in collaboration with the United States Public Health Service.

**Table 2:** Classification of anti-tuberculosis (anti-TB) drugs according to the site of action and its respective mechanism of resistance.

**Drug classification**	**Groups**	**Anti-TB drugs**	**Mechanism of action**	**Target TB**	**Efficacy**	**Mechanism of resistance**	**References**
Cell envelope synthesis inhibitor	Peptidoglycan	Cycloserine	Inhibit 2 enzymes forming D-alanine residues	MDR, XDR	High	Mutations in *alrA*	Patel et al., 2012
		Terizidone	Cycloserine derivative	MDR, XDR	High	Non	Galietti et al., 1991
	Arabinogalactan	Ethambutol	Inhibiting arabinosyltransferase, arabinose acceptor	Active	Low	*embCAB* operon	Telenti et al., 1997; Wolucka, 2008
	Mycolic acid	Isoniazid	Activation katG enzyme and inhibits inhA gene	Active, latent	High	Mutations in *katG* and *inhA* genes	Vilche‘ze and Jacobs, 2007; Riccardi et al., 2009
		Triclosan	Inhibits the inhA enzyme without activation katG	MDR	Low	–	Wang et al., 2004; Freundlich et al., 2009
		Pyridomycin	Inhibits the inhA enzyme	MDR, XDR	High	Mutations in *inha*	Hartkoorn et al., 2012
		Ethionamide	Inhibits InhA by enzyme ethA	MDR, XDR	High	Mutations in *inha, etha*	Wolff and Nguyen, 2012
		Prothionamide	Inhibits InhA by enzyme ethA	MDR, XDR	High	Mutations in *etha*	Wang et al., 2007
		Thiocarlide	Inhibiting synthesis of oleic acid	MDR, XDR	High	Mutations in *ethA*	Phetsuksiri et al., 2003
		Delamanid	Releasing Nitric oxide by Ddn enzyme	Active, latent, MDR, XDR	High	Non	Gler et al., 2012; Zhang et al., 2013
		SQ109	Membrane transporter *MmpL3*	Active, latent	High	Non	Owens et al., 2013
Protein synthesis inhibitor	Aminoglycosides	Streptomycin	Bind to 30S subunit of ribosome	MDR, XDR	High	Mutation in *rpsl*	Honort and Cole, 1994
		Amikacin	Bind to 30S subunit of ribosome	MDR, XDR	High	Mutation in *rrs*	Sowajassatakul et al., 2014
		Kanamycin	Bind to 30S subunit of ribosome	MDR, XDR	High	Mutation in *rrs*	Sowajassatakul et al., 2014
	Oxazolidone	Linezolid	Bind to 50S subunit of ribosome	MDR, XDR	High	Mutation in *G2576T*(23S)	Scheetz et al., 2008
		Sutezolid	Bind to 50S subunit of ribosome	MDR, XDR	High	Non	Zumla et al., 2015
	Peptidoglycan	Capreomycin	Peptidoglycan Breakdown	MDR, XDR	High	Mutation in *tlyA*	Chen et al., 2003
Nucleic acid inhibitor	Rifamycins	Rifampicin	RNA polymerase inhibitor	Active, latent	Low	Mutations in *rpoB* gene	Sensi, 1983; Telenti et al., 1993
		Rifapentine	RNA polymerase inhibitor (b-subunit)	Active, latent/HIV	High	Mutations in *rpoB* gene	Chan et al., 2014
		Rifabutin	RNA polymerase inhibitor (b-subunit)	Active, latent/HIV	High	Mutations in *rpoB* gene	Yan et al., 2015
		Rifalazil	RNA polymerase inhibitor (b-subunit)	Active, latent/HIV	High	Mutations in *rpoB* gene	Saribaş et al., 2003
	PAS	PAS	Folic acid synth inhibitor	MDR, XDR	High	Mutations in *thyA*	Patel et al., 2012
	Quinolones	Levofloxacin	DNA gyrase inhibitor	MDR, XDR	High	Mutations in *gyrA*	Pranger et al., 2011
		Moxifloxacin	DNA gyrase inhibitor	MDR, XDR	High	Mutations in *gyrA*	Pranger et al., 2011
New drugs		Bedaquiline	Inhibiting ATP synthase enzyme	Active, MDR, dorment,XDR	High	Mutations in *atpE* gene	Chan et al., 2013; Chahine et al., 2014
		Pyrazinamide	Interferes with binding to mRNA	Active,MDR	High	Mutations in *RpsA, pncA*	Zhang et al., 2003; Shi et al., 2011
		Clofazimine	Inhibits DNA replication	MDR, XDR/HIV	High	Mutations in *rv0678*	Arbiser and Moschella, 1995

*PAS:para-aminosalicylic acid*.

Latent TB has been treated usually with a single antibiotic to prevent progressing to active TB disease (Menzies et al., 2011), while active TB is now treated with combinations of antibiotics in order to reduce the growing risk of antibiotic resistance (Lawn and Zumla, 2011). Directly observed therapy-short course (DOTS) is currently recommended by the WHO as an effort to reduce the number of people not appropriately taking antibiotics (Volmink and Garner, 2007; Liu et al., 2008; Mainous and Pomeroy, 2010). When MDR-TB is detected, treatment with at least four effective antibiotics for 18–24 months is recommended (Lawn and Zumla, 2011). A person with fully-susceptible MTB may develop secondary resistance because of inadequate therapy, or using low-quality medication (O'Brien, 1994).

## Drug resistance for TB

More than 50% of the world's MDR-TB cases are found in India and China, where about 5.4% of MDR-TB cases progress to XDR-TB (WHO, 2010). The MDR-TB treatment is a combination of 8–10 drugs for 18–24 months (Gandhi et al., 2010). Resistance to the two most effective first-line anti-TB drugs, rifampicin and isoniazid, is known as MDR-TB, while resistance to three or more of the six classes of second-line drugs is known as XDR-TB (Table 1; CDC, 2006); the latter has been identified in more than 90% of the world's countries (Akachi et al., 2012). Total drug-resistant to all currently used drugs (McKenna, 2012) was first observed in Italy (2003) (Migliori et al., 2007), and had also been reported in Iran and India (Velayati et al., 2009; Akachi et al., 2012), but not widely reported until 2012 (WHO, 2006; Migliori et al., 2007).

MTB strains undergo spontaneous mutations that lead to resistance of one or more anti-TB drug (David, 1970). Thus, the exposure of MTB population to a single anti-TB drug could inhibit its growth but not completely eradicate it, therefore, regrowth and mutations leading to progressive drug resistance, these mutated genes are eventually triggering continuous proliferation of the bacilli and recurrence of symptoms, which is called “the fall and rise phenomenon” (Espinal, 2004). Hence, low drug levels due to insufficient drug bioavailability or from malabsorption (e.g., in HIV patients) have emerged in the etiology and mechanism of anti-TB drug resistance. Furthermore, continuous use of old anti-TB regimens may not target specific populations of MTB under certain circumstances that hardly act in acidic, or hypoxic conditions within caseous foci or inside macrophages (Mitchison, 1998). The resistance also developed independently for each drug in combined anti-TB regimens at a specific time through mutation processes.

We now know that MDR and XDR-TB infections' danger can be overcome by preventing resistance of already sensitive anti-TB drug within the combination regimen (Morris et al., 1995). As some studies reported, the resistance arises from replicating bacilli, while non-replicating bacilli do not undergo mutation and no resistance can be developed. Thus, minimizing the drug resistance can be performed by extending the therapeutic duration and subjecting MTB to drugs with longer half-lifes (Gumbo et al., 2009). Some newest agents that are used for MDR-TB, such as bedaquiline, are tentatively recommended (Chahine et al., 2014). Ineffective and inadequate anti-TB treatment could fail to achieve goals in about 30% of MDR-TB patients (Mitnick et al., 2003). The treatment of XDR-TB is very difficult, because XDR-TB bacilli are resistant for more drugs other than isoniazid and rifampicin, including fluoroquinolones and aminoglycosides (Ma et al., 2010).

The drug-resistant TB can be predicted in TB patients with unsuccessful therapy (relapse) or those who are in close contacts with MDR-TB patients (Becerra et al., 2011). Therefore, a 5 month treatment with positive sputum smear or culture is closely attributed to MDR-TB strains (Lew et al., 2008), and in such cases, several molecular methods for diagnosis of MDR-TB are enrolled, including the Xpert MTB/RIF, which is currently available for the detection of rifampicin resistance (Menzies et al., 2009; Sharma et al., 2015).

The last two decades have witnessed an ongoing effort to understand the molecular bases for anti-TB resistance and to further investigate the genetic traits in MDR- and XDR-TB strains (Nachega and Chaisson, 2003). Mutated genes that associated with MDR- and XDR-TB are described in Table 2, which are classified as first line anti-TB drugs resistance, that starts with isoniazid resistance; the latter is connected to alterations in the catalase-peroxidase gene (katG), the inhA gene, which encodes in an enzyme involved in mycolic acid biosynthesis (Vilche‘ze and Jacobs, 2007; Riccardi et al., 2009). Rifampin resistance, including its derivatives (rifapentine, rifabutin and rifalazil) resistance, is associated with genetic mutations in rpoB, which encodes the RNA polymerase β-subunit (Sensi, 1983; Telenti et al., 1993; Saribaş et al., 2003; Chan et al., 2014; Yan et al., 2015). Pyrazinamide resistance is linked to mutations in pncA, that eliminates the pyrazinamidase/nicotinamidase activity (Zhang et al., 2003; Shi et al., 2011). Ethambutol resistance is conferred to genetic mutations with the embCAB operon, which facilitates production of arabinosyl transferase (Telenti et al., 1997; Wolucka, 2008). In spite of the role of the second line drugs to overcome the MDR that linked with the first line drugs, second line drugs are also linked with genetic mutations like the first line agents: streptomycin resistance which is associated with mutations in the rpsL, ribosomal S12 protein, and rrs, 16S rRNA gene (Honort and Cole, 1994); kanamycin and amikacin resistance are closely linked to genetic mutations of streptomycin (Sowajassatakul et al., 2014); while capreomycin resistance is attributed to mutagenesis of the tlyA gene, which has homology to rRNA methyltransferases (Chen et al., 2003). Quinolones resistance (like levofloxacin and moxifloxacin) is associated with mutation of gyrA gene encoding DNA gyrase (Pranger et al., 2011). Ethionamide resistance is linked to inhA mutations, in addition to cross-resistance between isoniazid and ethionamide in mutations of the etaA (ethA) gene, which is responsible for ethionamide activation (Wolff and Nguyen, 2012). The resistance to PAS is linked to mutations within the thyA gene, which produces thymidylate synthase A (Patel et al., 2012), while cycloserine resistance is conferred with activation of the alrA gene as D-alanine racemase encoding, which causes increased over expression of alrA (Chacon et al., 2002).

Advances in MTB targeting have emerged through the exploration of the genome sequence of MTB (Cole et al., 1998), but unfortunately this approach gave little success (Payne et al., 2007) as it is not predicting the drug ability of the discovered new agent (Working Group on New TB Drugs, 2010). Genome sequencing of MTB, identification of the essential signaling and metabolic pathways, assessment of physicochemical properties of the MTB and other methods are still employed in order to discover newer agents with high specificity and less toxicity with good efficacy. In parallel, reengineering and repositioning of the old known drugs have been adapted to achieve better results in therapy, but the challenges of the resistance still threaten this goal and the discovering of the new agents remain the main approach to counteract the deterioration in situation over the world (Koul et al., 2011).

## Immunomodulatory and repurposing drugs against TB

An efficient and competent host immune system is crucial for the eradication of an MTB infection and/or containment of latent TB infection (Migliori and Huggett, 2009; Zumla et al., 2012; Wallis et al., 2013). The stability of latent TB state is achieved by MTB ability to attenuate and evade host mycobactericidal responses. Inadequate immunity leads to MTB multiplication and clinical symptoms' development. Acceleration of the host inflammatory response may lead to tissue destruction; therefore, several agents are being used in order to manipulate and reduce the destructive inflammatory responses, or augment protective immunity to enhance recovery and minimize the duration of therapy (Subbian et al., 2011; Tobin et al., 2012).

In experimental animals, the role of pro-inflammatory and anti-inflammatory eicosanoids in the process of regulating tumor necrosis factor-α levels (Tobin et al., 2012) and in tailoring TB treatment, depends on the host genotype (Skerry et al., 2012). Administered prophylactically or therapeutically, the ABL family tyrosine kinase inhibitor, such as imatinib, reduced the MTB load and the granulomatous lesions in MTB-infected organs and was also effective against a rifampicin-resistant strain of MTB when co-administered with current first-line TB drugs (Napier et al., 2012). Furthermore, using generic, non-steroidal anti-inflammatory (NSAIDs) and analgesic drugs as an adjunct therapy in experimental animal models, has a wide clinical distributed (Ivanyi and Zumla, 2013). NSAIDs can reduce MTB load and alleviate lung damage in mice (Vilaplana et al., 2013), and they show anti-TB activity in phenotypical assays (Guzman et al., 2013).

Both verapamil (a calcium-channel blocker) and reserpine (an adrenergic neuron blocking agent) have efflux pump inhibitory properties that could decrease macrophage-induced drug tolerance (Amaral et al., 2007; Adams et al., 2011); as a result, both could be added to anti-TB regimen to decrease the duration of curative therapy. Ivermectin is an anti-nematode agent that also has bactericidal activity against MTB (Lim et al., 2013). Cilostazol and sildenafil—as phosphodiesterase inhibitors—could be added to the anti-TB regimen, as they improve the resolution of tissue pathology, accelerating MTB clearance and diminishing therapeutic period (Maiga et al., 2012). Lansoprazole, a well-known proton-pump inhibitor, was also found to be effective against intracellular MTB by targeting its cytochrome bc1 complex through intracellular sulfoxide reduction (metabolite enzyme) to lansoprazole sulfide; this metabolite enzyme is crucial for the bacterium to produce energy, thereby killing it off (Rybniker et al., 2015). Metformin, which is a drug used for the treatment of type 2 diabetes, acts as inhibitor to a mitochondrial complex which is similar to bacterial NDH complex, thus enhancing the targeting of an anti-TB drug toward intracellular MTB (Cole et al., 1998; Vashisht and Brahmachari, 2015). Finally, chemical and biological immunomodulatory agents have also been evaluated to accelerate host immune responses in anti-TB therapy (Uhlin et al., 2012), with MDR-TB cure rate enhancement, prevention of recurrence and shortening therapy duration occurring as a result.

## TB/HIV co-infection

It is known that the concomitant use of anti-retroviral therapy (ART) with the treatment of drug-susceptible pulmonary TB improves survival rates in HIV-infected individuals. However, treatment of TB in such patients is complicated, due to potential drug interactions and the risk of developing “immune reconstitution inflammatory syndrome” (Gengiah et al., 2011). The important drug interactions occur between the rifamycins and the protease inhibitors as well as non-nucleoside reverse transcriptase inhibitor drugs. Rifamycin derivatives (rifampicin, rifabutin and rifapentine) induce liver enzymes and reduce serum concentrations of protease inhibitors, such as indinavir, nelfinavir, saquinavir, ritonavir, amprenavir, atzanavir, and fosamprenavir. Rifabutin is the least potent inducer of CYP3A (Weber et al., 2001) and rifapentine falls in between rifampicin and rifabutin in its capacity to induce CYP3A. Rifapentine is not recommended for the treatment of TB in HIV-infected individuals because of the increased rate of acquired rifamycin resistance (Dheda et al., 2010). Rifabutin is used as a substitute for rifampicin in the treatment of active TB in patients receiving ART. On the other hand, delaying initiation of ART until TB treatment is completed in HIV-infected individuals significantly increases mortality across the spectrum of immunodeficiency. Clinical trials have reported that early ART in TB patients co-infected with HIV decreases mortality (Havlir et al., 2011). The World Health Organization recommends that ART should be started within the first 8 weeks of initiating TB treatment (Blanc et al., 2011; De Cock and El-Sadr, 2013), while the optimal timing of initiating ART in patients with TB-HIV co-infection in Sub-Saharan Africa remains an urgent research priority (De Cock and El-Sadr, 2013).

## Prevention

The prevention and control of TB depend primarily on vaccination of infants and appropriate diagnosis and treatment of active cases (Lawn and Zumla, 2011). The US Preventive Services Task Force (USPSTF) recommends screening high risk people for latent TB with either tuberculin skin tests or interferon-gamma release assays (Bibbins-Domingo et al., 2016). The only available vaccine since 1921 is BCG (McShane, 2011). In children, BCG decreases the risk of getting the infection by 20% and the risk of infection turning into disease by nearly 60% (Roy et al., 2014). It is the most widely used vaccine worldwide, with more than 90% of all children being vaccinated (Lawn and Zumla, 2011). However, it should be noted that the immunity induced by the vaccine decreases after about 10 years (Lawn and Zumla, 2011). Moreover, as TB is uncommon in most of Canada, the UK, and the USA; BCG is administered only to those at high risk (CDC, 2006; Teo and Shingadia, 2006; Public Health Agency of Canada, 2010). Finally, the drawback of the BCG vaccine is making the tuberculin skin test result false positive; therefore, this test not widely used in screening for TB anymore (Teo and Shingadia, 2006).

## Author contributions

All authors listed have made a substantial, direct and intellectual contribution to the work, and approved it for publication.

### Conflict of interest statement

The authors declare that the research was conducted in the absence of any commercial or financial relationships that could be construed as a potential conflict of interest.
